# Gene Loss, Pseudogenization in Plastomes of Genus *Allium* (*Amaryllidaceae*), and Putative Selection for Adaptation to Environmental Conditions

**DOI:** 10.3389/fgene.2021.674783

**Published:** 2021-07-08

**Authors:** Victoria A. Scobeyeva, Ilya V. Artyushin, Anastasiya A. Krinitsina, Pavel A. Nikitin, Maxim I. Antipin, Sergei V. Kuptsov, Maxim S. Belenikin, Denis O. Omelchenko, Maria D. Logacheva, Evgenii A. Konorov, Andrey E. Samoilov, Anna S. Speranskaya

**Affiliations:** ^1^Department of Evolution, Faculty of Biology, Lomonosov Moscow State University, Moscow, Russia; ^2^Department of Molecular and Biological Physics, Moscow Institute of Physics and Technology, Dolgoprudny, Russia; ^3^Department of Vertebrate Zoology, Faculty of Biology, Lomonosov Moscow State University, Moscow, Russia; ^4^Department of Higher Plants, Faculty of Biology, Lomonosov Moscow State University, Moscow, Russia; ^5^Faculty of Bioengineering and Bioinformatics, Lomonosov Moscow State University, Moscow, Russia; ^6^Botanical Garden, Faculty of Biology, Lomonosov Moscow State University, Moscow, Russia; ^7^Laboratory of Plant Genomics, Institute for Information Transmission Problems, Moscow, Russia; ^8^Center of Life Sciences, Skolkovo Institute of Science and Technology, Moscow, Russia; ^9^Laboratory of Animal Genetics, Vavilov Institute of General Genetics, Russian Academy of Science (RAS), Moscow, Russia; ^10^Group of Genomics and Postgenomic Technologies, Central Research Institute of Epidemiology, Moscow, Russia

**Keywords:** *Allium*, plastome, sequence, evolution, pseudogenization

## Abstract

*Amaryllidaceae* is a large family with more than 1,600 species, belonging to 75 genera. The largest genus—*Allium*—is vast, comprising about a thousand species. *Allium* species (as well as other members of the *Amaryllidaceae*) are widespread and diversified, they are adapted to a wide range of habitats from shady forests to open habitats like meadows, steppes, and deserts. The genes present in chloroplast genomes (plastomes) play fundamental roles for the photosynthetic plants. Plastome traits could thus be associated with geophysical abiotic characteristics of habitats. Most chloroplast genes are highly conserved and are used as phylogenetic markers for many families of vascular plants. Nevertheless, some studies revealed signatures of positive selection in chloroplast genes of many plant families including *Amaryllidaceae*. We have sequenced plastomes of the following nine *Allium* (tribe *Allieae* of *Allioideae*) species: *A. zebdanense*, *A. moly*, *A. victorialis*, *A. macleanii*, *A. nutans*, *A. obliquum*, *A. schoenoprasum*, *A. pskemense*, *A. platyspathum*, *A. fistulosum*, *A. semenovii*, and *Nothoscordum bivalve* (tribe *Leucocoryneae* of *Allioideae*). We compared our data with previously published plastomes and provided our interpretation of *Allium* plastome genes’ annotations because we found some noteworthy inconsistencies with annotations previously reported. For *Allium* species we estimated the integral evolutionary rate, counted SNPs and indels per nucleotide position as well as compared pseudogenization events in species of three main phylogenetic lines of genus *Allium* to estimate whether they are potentially important for plant physiology or just follow the phylogenetic pattern. During examination of the 38 species of *Allium* and the 11 of other *Amaryllidaceae* species we found that *rps16*, *rps2*, *infA*, *ccsA* genes have lost their functionality multiple times in different species (regularly evolutionary events), while the pseudogenization of other genes was stochastic events. We found that the “normal” or “pseudo” state of *rps16*, *rps2*, *infA*, *ccsA* genes correlates well with the evolutionary line of genus the species belongs to. The positive selection in various NADH dehydrogenase (*ndh*) genes as well as in *matK*, *accD*, and some others were found. Taking into account known mechanisms of coping with excessive light by cyclic electron transport, we can hypothesize that adaptive evolution in genes, coding subunits of NADH-plastoquinone oxidoreductase could be driven by abiotic factors of alpine habitats, especially by intensive light and UV radiation.

## Introduction

Vascular plants inhabit various ecology niches which may be distinguished by sets of environmental factors (e.g., irradiation level, atmospheric and soil humidity, temperature), all of them may affect photosynthesis. Mountain altitudinal gradient as well as redundantly lit or (as opp.) constantly shadowed habitats seem to be the most powerful “natural geophysical” pressure for evolutionary modification of genes of photosynthetic apparatus. The genes (approximately 120–130 genes) present in chloroplast genomes (plastomes) encode the core proteins of photosynthetic complexes: Photosystem I (*psaA*, *B*, *C*, *I*, and *J*) and Photosystem II (*psbA-F*, *H-N*, *T*, and *Z*), Cytochrome b6f (*petA*, *B*, *D*, *G*, *L*, and *N*)[Hu et al., 2015), NADH dehydrogenase (*ndhA-K*), ATP synthase (*atpA*, *B*, *E*, *F*, *H*, and *I*), the large RUBISCO subunit (*rbcL*), chloroplast ribosomal proteins of large and small subunits (*rpl* and *rps*), polymerase subunits (*rpoA*, *B*, *C1*, and *C2*), ATP-dependent protease (*clpP*), cytochrome c biogenesis (*ccsA*), membrane protein (*cemA*), translation initiation factor I (*infA*), maturase (*matK*) and some proteins of known or unknown functions of (*ycf1_short*, *ycf1_long*, *ycf2*, *ycf3*, *ycf4*), as well as four ribosomal RNAs and various tRNAs (Wicke et al., 2011; Daniell et al., 2016). The core set of plastom’s genes is retained from cyanobacteria ancestors; most of them are required for the light reactions of photosynthesis or functions connected with transcription and translation (Sanchez-Puerta et al., 2005; Green, 2011). The evolution of plastome genes could be under pressure of geophysical abiotic factors of plant habitats (Hanaoka et al., 2012; Hu et al., 2015; Zhang et al., 2016; Macadlo et al., 2020). Among geophysical abiotic factors species vertical range limits seem to be more niche-dependent than horizontal (Hargreaves et al., 2014) and vertical limits are often species niche limits (Lee-Yaw et al., 2016).

Species adaptations to diverse environments are accompanied by mutations under positive selection which may be confirmed using analysis of Kn/Ks ratio. Numerous genes were proved to be under positive selection in taxons which are myco-heterotrophic plants, and also in plants that are partly or entirely non-photosynthetic (Zeng et al., 2017; Dong et al., 2018). The sets of genes which are under positive selection in plants with adaptation to various ecological niches seem to be incoherent. Positive selection in some genes, e.g., *rbcL*, is widespread in most lineages of land plants (Yao et al., 2019). Some authors propose they are associated with adaptation to various ecological niches, including dry or wet habitats (Kapralov and Filatov, 2006), high altitudes in mountain regions (Hu et al., 2015). *matK* is reported to be a highly variable sequence which was often used for phylogeny analysis of many plant taxons (Hilu et al., 2003; Tamura et al., 2004). Different sets of genes are found under positive selection in species of various taxonomic groups. For example, in *Oryza* species (*Poaceae*, *Monocotyledones*) which were adapted to either shady or sunny environments, a set of positively selected genes (besides *rbcL* and *matK*) was found: *accD*, *ndhD*, *ndhF*, *ndhH*, *psaA*, *psbB*, *psbD*, *psbH*, *rpl16*, *rpoA*, *rpoC2*, and *ycf68* (Gao et al., 2019). A completely different set of genes (*ycf1*, *ycf2*, *rps14*, *rps15*, and *rps16)* was found to be under selection in *Cardamine* and *Nasturtium* species (*Brassicaceae*) (Yan et al., 2019). Another analysis of adaptive evolution in *Brassicaceae* plastomes in general and in the *Cardamine* genus in particular resulted in detection of signatures of positive selection in the following genes (besides *rbcL* and *matK*): *ycf1*, *rpoC2*, *rpl14*, *petD*, *ndhF*, *ccsA*, *accD*, and *rpl20* at a significant level (Hu et al., 2015). Presumably, detected signals of positive selection in genes may reflect specific adaptations of species to a particular habitat. For example, in *Brassicaceae* it could possibly be a consequence of adaptation to high altitude environments (Hu et al., 2015).

Furthermore, extensive structural changes, such as large inversions, deletions, loss of functionality (pseudogenization or complete loss) of genes are regularly observed in plastomes of certain plant species or groups of species (various taxa of higher rank) (Haberle et al., 2008; Guisinger et al., 2011; Schwarz et al., 2015; Hsu et al., 2016; Fonseca and Lohmann, 2017). For example, the loss of all *ndh* (NADH dehydrogenase subunit complex) genes is apparently a result of convergent evolution during the land adaptation of photosynthesis in neighboring clades of different taxa. The *ndh* genes loss in plastomes is usually linked to heterotrophic type of nutrition, like in *Orchidaceae* (Lin et al., 2015; Lin et al., 2017) or *Lentibulariaceae* (Silva et al., 2016). However, the *NDH* complex may also be lost in photoautotrophic plant species, as it was discovered for a wide spectrum of angiosperms and gymnosperms (de Pamphilis and Palmer, 1990; Haberhausen and Zetsche, 1994; Wakasugi et al., 1994; Martín and Sabater, 2010; Blazier et al., 2011; Omelchenko et al., 2020). Presumably, the loss of *ndh* genes is linked with inability to tolerate light intensity stress (Omelchenko et al., 2020). But for most plastome genes implications of their loss on the physiological and ecological functionality of plants remain inexplicable and are interpreted as random evolution events or just stated as fact. For example, *rpl23* has been found to be a pseudogene in monocots (Ogihara et al., 1991; Morton and Clegg, 1993; Omelchenko et al., 2020) and in all species of *Caryophyllales* (eudicots) (Raman and Park, 2015), while a wide range of species belonging to other clades of eudicots (e.g., rosids, asterids) have no traces of pseudogenization of *rpl23* (Raman and Park, 2015).

*Amaryllidaceae* is a large family with more than 1,600 species, divided into three subfamilies: *Agapanthoideae*, *Amaryllidoideae*, and *Allioideae*. The genus *Allium* (*Allioideae*) is vast, being one of the largest monocotyledonous genera and comprising more than 800 species (Fritsch et al., 2010; Fritsch, 2012; Shah, 2014; Maragheh et al., 2019) or about 973 species according to WCSP database (Govaerts and Fritsch, 2021). *Allium* species form 15 subgenera that are grouped into three intrageneric evolutionary lineages according to molecular data analysis of plastid and nuclear DNA barcode sequences (Friesen et al., 2006; Li et al., 2010; Wheeler et al., 2013). Besides the *Allium* genus (comprising monogeneric tribe *Allieae*), *Allioideae* has 17 more genera belonging to another three tribes (*Gilliesieae*, *Leucocoryneae*, and *Tulbaghieae*) (Chase et al., 2009; Sassone et al., 2014). *Allium* species (as well as other members of the *Amaryllidaceae*) are widespread and diversified, they are adapted to a wide range of habitats from shady forests to open habitats like meadows, steppes and deserts and highlands and are therefore well suited as study objects in studies of plants’ adaptations to various sun irradiation levels and other geophysical factors (Vvedensky, 1935; Wendelbo, 1971). Complete plastome sequences were first sequenced and arranged for the most economically important species of *Allium*: onion, *A. cepa* (von Kohn et al., 2013), garlic, *A. sativum* (Filyushin et al., 2016), edible species *A. ursinum* and *A. paradoxum* (Omelchenko et al., 2020). Another wide spectrum of wild and cultivated *Allium* species plastomes were sequenced and partially analyzed (Filyushin et al., 2018, 2019; Xie et al., 2019, 2020; Liu et al., 2020; Namgung et al., 2021).

According to data available, plastomes of most plant species contain the same number of tRNA genes (30 in total, of them 9 are represented by two copies in IR) and rRNA genes (eight genes, all four are represented by two copies in IR), while the rest 79 genes encode proteins (Wicke et al., 2011; Wambugu et al., 2015). Investigated *Allium* species had similar number, arrangement and orientation of genes (Filyushin et al., 2016, 2018; Huo et al., 2019; Yusupov et al., 2020). Only *A. paradoxum* demonstrated notable alterations, such as large 4,825 bp long local inversion in the SSC region and elimination or pseudogenization of the whole *ndh* gene family, as well as large number of other genes: *rps16*, *rps2*, *rpl22*, *petD*, *infA*, *rpl23*, *rps3* (Omelchenko et al., 2020). Recently positive selection pressure was calculated in subgenus *Anguinum* of *Allium* and three genes were found with Kn/Ks >1 (*accD*, *rps14*, *rpl33*) (Jin et al., 2019). Soon after this an analysis of average Kn/Ks in a total of 39 complete chloroplast genomes of *Allium* was performed and quite unexpectedly it revealed an absolutely different set of positively selected genes (with Kn/Ks > 1): *psbC*, *rps11*, and *psaI* (Xie et al., 2020). These inconsistent data prompted us to choose a simple and easy to analyze ecological trait—the highest tolerable altitude of the species. High altitude habitats have high levels of solar radiation, often low humidity and other special meteorological conditions, influencing the whole plant physiology (Gale, 2004). We took the highest tolerable altitude of the *Allium* species as a selective factor and performed an evolutionary analysis of plastome genes in species with contrasting altitude limits (see Supplementary Material 2 for species list). In this manuscript we only report results that relate to protein-coding genes of examined plastomes. We report *de novo* sequencing and assembling of complete cp-genomes of wild and cultured *Allium* species as well as outgroup species belonging to the same subfamily of *Allioideae* in *Amaryllidaceae*—*Nothoscordum bivalve* (L.) Britton. We have identified rapidly evolving plastome regions and genes under positive selection, and detected plastome traits that differentiate between species of three evolutionary lines of *Allium* genus adapted to certain contrasting habitat types: high-altitude vs. lowland habitats.

## Materials and Methods

### Sampling, DNA Extraction, cpDNA Isolation, and Sequencing

Plastomes were newly sequenced and assembled for the following species obtained alive from the outdoor collection of the Moscow State University Botanical Garden: *A. fistulosum* L., *A. macleanii* Baker, *A. nutans* L., *A. obliquum* L., *A. pskemense* B. Fedtsch., *A. schoenoprasum* L., *A. victorialis* L., *A. zebdanense* Boiss. & Noë. The specimens of *A. semenovii* Regelas well as *A. platyspathum* Schrenk were collected in the wild in 2016. The specimens of the abovementioned species from MSU Botanical Garden living collections and from wild natural habitats were deposited in the Moscow State University Herbarium, extracted DNA from *Allium* plants were deposited in DNA collection in MSU Biology Department. The plastomes of *A. moly* L and *Nothoscordum bivalve* (L.) Britton were sequenced using dry plant material obtained from Osnabrück Botanical Garden (Germany), kindly provided by Nikolai Friesen. All additional details, including taxonomic position of species used in the study, the source of specimens, GenBank/ENA accession numbers of plastome sequences, information about ecology and geographical location of wild collected specimens, accession number of vouchers are provided in Supplementary Table 2.

For cpDNA extraction from samples obtained from living specimens (Lomonosov MSU botanical garden collections),fresh leaves were cut and stored in the dark in a refrigerator at +4°C for 10 days. Chloroplasts were isolated from about 2 g (fresh weight) of leaves using protocol based on Shi et al. (2012) and Vieira et al. (2014) and described in detail in Logacheva et al. (2017). Then cpDNA was extracted using standard CTAB protocol (Doyle and Doyle, 1987). Quality of cpDNA was evaluated visually by gel-electrophoresis.

Another approach was used for herbarium samples of *A. semenovii*, *A. platyspathum*, *A. moly*, and *N. bivalve.* Fragments of dry leaves were used as material for total DNA extraction according to Doyle and Doyle (1987) with an additional stage of purification according to Krinitsina et al. (2015) because of high amount of impurities (presumably phenolic compounds). Quality of total DNA was evaluated using spectrophotometry (Nanophotometr-N-60, Implen). Quantification of nucleic acids was done using fluorimetry (Qubit 3.0, Thermo Fisher Scientific).

### Library Preparation, Sequencing, and Data Assembling

The libraries with insert sizes of 400–800 bp were constructed and then sequenced using high-throughput sequencing platform Illumina MiSeq (PE 2 × 250 bp or 2 × 300 bp). Paired-end libraries for each DNA sample were constructed at least twice using two different library preparation strategies to minimize protocol-associated biases and maximize assembly efficiency. First strategy included physical fragmentation by Covaris 220 followed by protocol with adapter ligation, using NEBNext^®^ DNA Library Prep Master Mix Set for Illumina (E6040, NEB reagents) with single indexed primers from NEBNext^®^ Multiplex Oligos for Illumina Kits (Index Primers Set 1–4), used according to the manufacturer’s recommendation. Another approach was DNA library preparation using a transposase-based method (Nextera), developed by Illumina. After tagmentation the libraries were amplified using NEB Q5^®^ High-Fidelity DNA Polymerase (up to 12 cycles of amplification) and Nextera-compatible dual indexed primers.

The chloroplast genome assembly protocol included (1) quality trimming with Trimmomatic (Bolger et al., 2014), (2) filtering of reads using known chloroplast genome sequences of *A. cepa* (NC024813) and *A. sativum* (NC031829) by Bowtie2 mapper (Langmead and Salzberg, 2012), (3) producing of two contig sets for both filtered and non-filtered reads using *de novo* assemblers Velvet (Zerbino and Birney, 2008) and Spades (Bankevich et al., 2012). Assembled contigs were selected for the next assembly if they showed similarity to published *Allium* plastomes. The final *de novo* assembly was checked and fixed where necessary by PE reads mapping to the assembly in order to check for potential assembly artifacts using Bowtie2, VarScan (v.2.3.7) and SAMtools/BCFtools software packages (Li et al., 2009; Langmead and Salzberg, 2012; Koboldt et al., 2012). The coverage was as follows: *A. semenovii* ∼30×, *A. macleanii* ∼35×, *A. fistulosum* > 200×, *A. platyspathum* ∼35×, *A. nutans* > 300×, *A. obliquum* > 200×, *A. pskemense* ∼39×, *A. schoenoprasum* L. > 250×, *A. victorialis* ∼80×, *A. zebdanense* > 150×.

Plastomes of *A. macleanii*, *A. nutans*, *A. obliquum*, *A. platyspathum*, *A. pskemense*, *A. schoenoprasum*, *A. victorialis*., *A. zebdanense* were completely assembled using only the high-throughput sequencing approach. Some unassembled regions of *A. semenovii* plastome were sequenced by Sanger method. Primers for amplification were: **Alsem 1 for:** GTC CTC GGT AAC GAG ACA TAA; **Alsem 1 rev:** ACG TAG TCA ACT CCA TTC GT; **Alsem 2 for:** GTG CCC AAA ATG GTG TCA AT; **Alsem 2 rev:** ATC CAT GGT TTA TTC CTT ATC TCT; **Alsem 3 for:** GTA TGC CGT CTT CTG CTT G; **Alsem 3 rev:** AAG GGT TCT TTT AAA CTC TTT TGT T; **Alsem 4 for:** TGT TGG ACA ATA CTC GAC AC; **Alsem 4 rev:** GAC CAT AGA GGA GCC GTA TG; **Alsem 5 for:** GAG TGG AGC TAT ACC CAA TAG ATA; **Alsem 5 rev:** TAA GGT TAT CTC CCG CCA AT.

### Plastome Annotation

The Dual Organellar GenoMe Annotator (DOGMA) (Wyman et al., 2004) and GeSeq (Tillich et al., 2017) programs were used for preliminary gene annotation. From this initial annotation, putative start codons, stop codons, and intron positions were determined. Then putative start and stop codons together with intron positions were manually corrected based on comparisons with homologous genes of cp genomes of *Lycoris squamigera* (NC_040164.1), *Narcissus poeticus* voucher WSY: WSY0108940 (NC_039825.1), *Lycoris radiata* (NC_045077.1), *Allium cepa* (NC_024813.1), *Agapanthus coddii* voucher K:20081397 (NC_035971.1), *Allium paradoxum* (NC_039661), *Allium herderianum* (NC_042156.1), and *Allium victorialis* (NC_037240.1). All identified tRNAs were further verified by tRNAscan-SE 1.21 (Chan and Lowe, 2019). Complete nucleotide sequences of plastomes sequenced in this study were deposited in the GenBank database under accession numbers listed in Supplementary Table 1. After this results of annotations were manually checked to correct errors that appear as a result of the work of algorithms implemented in the annotation software.

### Plastome Features Comparison

To investigate the sets of protein coding genes that reflect the evolution of plastomes in the three main clades of the genus *Allium* (Friesen et al., 2006), we used all complete and partially assembled sequences and complete sequences of several other *Allium* species plastomes obtained from GenBank. All annotated sequences previously published in GenBank were re-annotated using in-house scripts and *A. cepa* (NC_024813) as basic reference sequences. *A. praemixtum* (NC_044412) and *N. poeticus* (NC_039825) were used for annotation of genes pseudogenized or absent in *A. cepa*. Then we excluded most cultivated species, besides taken as reference, to avoid possible bias due to unknown processes accompanying artificial selection of cultivated forms. The sequences of the protein coding genes were extracted, than extraction and translation were manually verified to correct possible errors that appear as a result of the work of algorithms implemented in the annotation software. Although a fairly large number of complete *Allium* plastome sequences are available online, taxonomic attribution of many species looks uncertain. We used the species that we sequenced and assembled during this project and added more plastomes from GenBank, choosing species that were important for the aims of our research, namely representatives of the first and the second evolutionary line (Friesen et al., 2006) with contrasting highest tolerated altitudes: *A. paradoxum* (M. Bieb.) G. Don (MH053150), *A. ursinum* L. (MH157875.1), *A. prattii* L. (NC_037432.1), two important agricultural species *A. cepa* L. (NC_024813), *A. sativum* L. (NC_031829), and two species with known intraspecific variability [*A. obliquum* (MG670111) and *A. victorialis* (MF687749)]. For the complete list of analyzed species see Supplementary Table 2.

### Detecting of Pseudogenes, GC-Content, Positive Selection, and Evolution Rate Analysis

Primary alignment was prepared with MAFFT v7.471 (Kazutaka and Standley, 2013). To identify pseudogenes, we aligned nucleotide sequences with MACSE and marked the first position of either frameshift, stop-codon or deletion spanning to the sequence end. Alignments were visualized with JalView v2.11.1.3 (Waterhouse et al., 2009). Indels-containing regions were inspected by eye for possible alignment errors. No additional alignments filtering was applied. Pseudogene heatmap was constructed with pandas (Reback et al., 2021), numpy (Harris et al., 2020), seaborn (Waskom et al., 2020), and matplotlib (Hunter, 2007) Python3 packages (Cock et al., 2009; Van Rossum and Drake, 2009).

We calculated GC content and proportion of gaps by window 100 bp in length using custom python script. For every window substitution count was estimated using a fixed tree topology (ParsimonyScorer class in Biopython v 1.78 (Talevich et al., 2012). Tree topology was the same as for HyPhy methods (explained below). Plots were constructed with ggplot2 R package (Wickham, 2016; R Core Team, 2018).

For selection and evolutionary rate analysis we used the concatenated sequences of 78 genes (see Supplementary Table 3). All individual gene sequences recognized as pseudogenes were replaced with gaps. Phylogenetic analysis was conducted with IQ-TREE v 2.0.6 (Chernomor et al., 2016; Minh et al., 2020). Evolutionary models and partitioning schemes were optimized using built-in methods (Kalyaanamoorthy et al., 2017) starting from individual partitions for every gene. The final scheme had seven partitions. Obtained tree was used for assessment of per-gene evolutionary rates and selective pressure detection. Tree was visualized with FigTree v1.4.4 (Rambaut and Drummond, 2012). We applied FUBAR v2.2 (Murrell et al., 2013), aBSREL 2.1 (Smith et al., 2015), and MEME 2.1.1 (Murrell et al., 2012) methods implemented in HyPhy framework v2.5.2 MP (Kosakovsky Pond et al., 2005). The latter was used to obtain Kn/Ks ratios for genes.

We have carried out analysis and identification of genes under positive or diversifying selection in *Allium* genus in its adaptive evolution to different solar irradiation and other abiotic conditions of its wide range in highlands and various lowlands in the temperate zone. A total of 49 species was analyzed, of which data for 13 species was obtained by our group. Two species sequenced by our group already had been stored in databases earlier (*A. obliquum* and *A. victorialis*), so we took 51 sequences in analysis total. We have chosen 8 species from the first evolutionary line, 7 from the second and 23 from the third. Cultivated species *A. cepa* was included in the analysis as a reference, *A. sativum*, *A. fistulosum*, *A. tuberosum*, *A. chinense* were excluded from the analysis. As far as species vertical limits are often species niche-limits (Hargreaves et al., 2014), we took the highest tolerable altitude of the species listed, using the data available from literature sources (see Supplementary Table 2) as well as the information presented on the herbarium labels for the corresponding species in the herbarium of the Moscow State University^1^. Our analysis consisted of a classical approach with Kn/Ks calculation, aBSREL (Smith et al., 2015), MEME (Murrell et al., 2012), and FUBAR (Murrell et al., 2013). We considered evidence of positive selection as sufficiently reliable only for those cases where it was confirmed by several methods.

To compare ratio of genes under selection among different gene ontologies we performed Fisher’s exact test for multiple samples. All genes, used for selection seeking, were divided into seven ontologies—ATP synthesis, Cytochrome complex and electron-transport chain, *ndh* genes, photosynthesis, translation, transcription and other. For all the ontologies were calculated ratio of sites under selection to sites without selection and compared with Fisher’s exact test for multiple samples with R package (Holm–Bonferroni method was used to counteract the problem of multiple comparison).

## Results

We have sequenced, assembled and annotated plastomes of *A. zebdanense* (MZ019480), *A. moly* (MZ019477), *A. victorialis* (NC_037240.1), *A. macleanii* (LT699703.1), *A. nutans* (LT799837.1), *A. obliquum* (MZ019478), *A. schoenoprasum* (LT699700.1), *A. platyspathum* (LT673892.1), *A. semenovii* (MZ019479), *A. pskemense* (MZ147623), *A. fistulosum* (LT674586.1). In addition, we have obtained a complete nucleotide sequence of the plastome of *Nothoscordum bivalve*. Together with genus *Allium* it belongs to the *Allioideae* subfamily, but to a different tribe *Leucocoryneae*. The plastome sequence of *Nothoscordum bivalve* was submitted as (MZ019481). The main parameters, i.e., total/LSC/SCC/IRs lengths and complete comparative annotations for most of assembled plastomes are represented in the (Supplementary Material 1). To expand the sampling, we took the plastome sequences of other wild *Allium* species published in GenBank. In total 41 plastome sequences of wild *Allioideae* species were analyzed. In addition our analysis also included representatives of two other *Amaryllidaceae* subfamilies, *Agapanthoideae* and *Amaryllideae*, data on which were found at Genbank. For the complete list of the studied species and descriptions of general ecology features of their natural habitats (see Supplementary Material 2).

**FIGURE 1:**
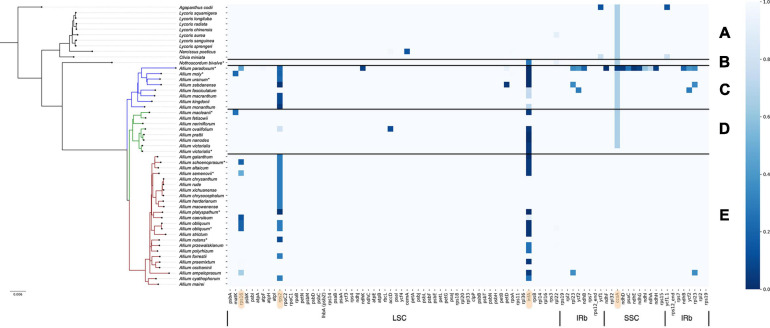
Pseudogenization or loss of protein-coding genes heatmap in the plastome of *Allioideae* (including all three evolution lines of *Allium*). Taxonomic position of species is denoted at the left side. Species sequenced by our group are marked with (*). **(A)** Shows *Agapanthoideae* and *Amaryllidoideae* subfamily species; **(B)** shows *Nothoscordum bivalve* as an outgroup for *Allium* genus; **(C)** shows the first evolutionary line *Allium* species; **(D)**—the second and **(E)**—the third. Genes are sorted in natural order in plastid. *LSC*, large single-copied region; *SSC*, small single-copied region; *IRa* and *IRb*, inverted repeats A and B. The darkest blue marks completely deleted genes; skyblue of varying shades marks pseudogenized genes (depending on the length of the preserved gene fragment); light blue marks true genes (translated without errors). Genes described below in detail are marked with peach color (*rps16*, *rps2*, *infA*, *ccsA*).

To identify and correct possible annotation errors that arise as a result of the work of algorithms implemented in annotation software, we performed automatical reannotation of all previously published plastome sequences that were selected for comparative analysis in this work, using GeSeq. In addition to this we had determined start and stop codons of all protein coding genes using in-house scripts. The results of both procedures were then manually compared and finalized. This step allowed us to find some noteworthy inconsistencies with previous reports, in particular, regarding pseudogenization status of *rps2*, *rps16*, and *ndhD* reported by Xie et al. (2019). The authors claim that *rps2* was lost in all *Allium* species (*Allioideae*), but we have found that pseudogenization occurred in only about a third of studied *Allium* species, while in the rest two thirds these genes proved to be in their true state, coding proteins without stop-codons (see Figure 1). Manual correction of automatic annotations has also influenced our conclusions regarding functionality of various genes like *rps16* in *A. platyspathum* and *A. nutans*: we found that these genes in these species are true. The *ndhD* gene also proved to be in its true state in all investigated *Allium* species (excluding *A. paradoxum*), in contradiction to conclusions made by Xie et al. (2019).

### Interrelation of Plant Preferences to Habitats/Phylogenetics and Deletion/Pseudogenization of Genes in Plastomes

The set of plastomes obtained were used for analysis of deleted/pseudogenized genes patterns and their association with evolutionary lineages and with environmental adaptation of species. Plastomes of *Allium* species as well as other known *Amaryllidaceae* are generally similar in composition of their functional gene sets. The main differences were found in the functionality of the following genes: *ccsA* (responsible for heme attachment to cytochromes c), *rps16* and *rps2* (proteins of the small ribosomal subunit), *infA* (translation initiation factor I), see Figure 1. Pseudogenization of some other genes is presumably a sporadic event in the *Allium* genus, sometimes happening in all evolutionary lines: *petD* (encodes a subunit of the cytochrome b6/f complex), *ycf1* (translocon on the inner plastid membrane), *ycf2* (encodes a subunit of the 2-MD heteromeric AAA-ATPase complex which associated with the TIC complex (Kikuchi et al., 2018) and some other genes can undergo occasional defunctionalization.

Undoubtedly the sequence features of the *rps2*, *infA*, *rps16*, *ccsA* genes as well as their “normal” or “pseudo” state correlate with the genus evolutionary line to which species belong to.

#### The *rps16* Gene Encoding Plastid 30S Ribosomal Protein S16

**FIGURE 2:**
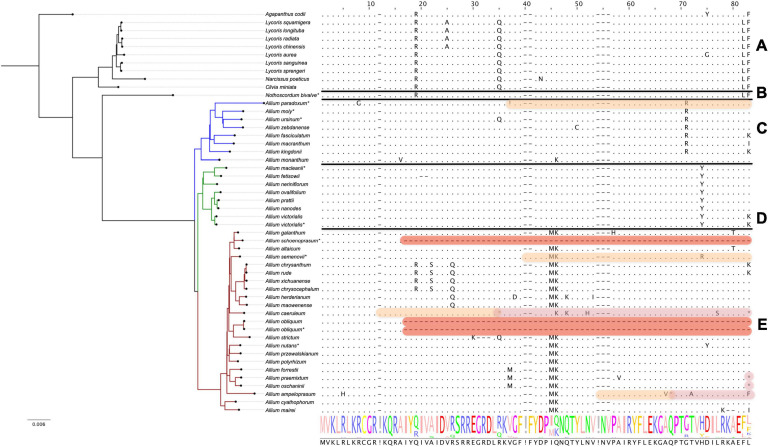
Protein sequence alignment of the plastid 30S ribosomal protein S16 encoded by *rps16* genes. Àll species are sorted by phylogenetic tree: the first evolutionary line of *Allium* is blue, the second is green, the third is red and the remaining species are black. Species sequenced by our group are marked with *. **(A)** Shows *Agapanthoideae* and *Amaryllidoideae* subfamily species; **(B)** shows *Nothoscordum bivalve* as an outgroup for *Allium* genus; **(C)** shows the first evolutionary line *Allium* species; **(D)**—the second and **(E)**—the third. Non-conserved AA positions are highlighted as letters and conserved as dots. The whole AA sequence is shown as a normalized alignment logo below. Possible frameshifts are shown as! and stop codons as *. Damaged sequences are highlighted in peach color in case of frameshift, in purple in case of stop codon and in red in case of total AA loss. Colored sequences may be assumed to be a pseudogene. The gene is absent in *A. platyspathum*.

We found that pseudogenization affects *rps16* genes only in species of the third evolutionary line, namely *A. schoenoprasum*, *A. obliquum*, *A. ampeloprasum*, *A. caeruleum*, and *A. platyspathum* (see Figure 2). In plastomes of first and second evolutionary lines of *Allium* (with only one exception, *A. paradoxum*) as well as in all non-*Allium Amaryllidaceae*, *rps16* gene is translated without errors.

#### The *rps2* Gene Encoding Plastid 30S Ribosomal Protein S2

**FIGURE 3:**
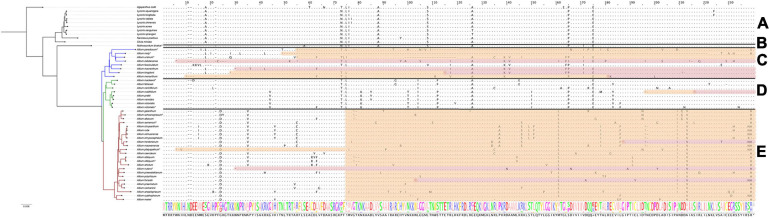
Protein sequence alignment of the plastid 30S ribosomal protein S2 encoded by *rps2* genes. Àll species are sorted by phylogenetic tree: the first evolutionary line of *Allium* is blue, the second is green, the third is red and the remaining species are black. Species sequenced by our group are marked with *. **(A)** Shows *Agapanthoideae* and *Amaryllidoideae* subfamily species; **(B)** shows *Nothoscordum bivalve* as an outgroup for *Allium* genus; **(C)** shows the first evolutionary line *Allium* species; **(D)**—the second and **(E)**—the third. Non-conserved AA positions are highlighted as letters and conserved as dots. The whole AA sequence is shown as a normalized alignment logo below. Possible frameshifts are shown as ! and stop codons as *. Damaged sequences are highlighted in peach color in case of frameshift and in purple in case of stop codon. Colored sequences may be assumed to be a pseudogene.

Defunctionalization of the *rps2* gene (encoding plastid 30S ribosomal protein S2) was found to be characteristic of most *Allium* species (see Figure 3). There is a considerable amount of single nucleotide substitutions along with several indels in the sequence of the *rps2* gene which leads to many non-synonymous AA substitutions and premature stop codons. Interestingly, at first glance, the species of the second evolutionary line seem to be less affected by this process, *rps2* being pseudogenized only in one species, *A. ovalifolium*.

#### The *infA* Gene Encoding Translation Initiation Factor I

**FIGURE 4:**
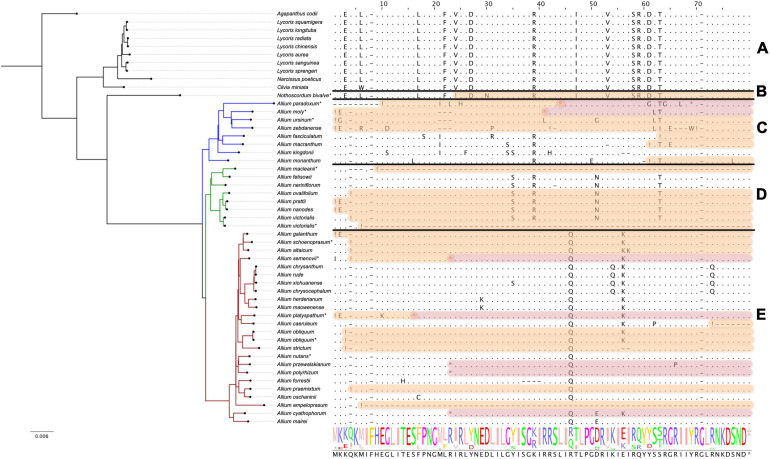
Protein sequence alignment of the translation initiation factor IF-1 encoded by *infA* genes. Àll species are sorted by phylogenetic tree: the first evolutionary line of *Allium* is blue, the second is green, the third is red and the remaining species are black. Species sequenced by our group are marked with *. **(A)** Shows *Agapanthoideae* and *Amaryllidoideae* subfamily species; **(B)** shows *Nothoscordum bivalve* as an outgroup for *Allium* genus; **(C)** shows the first evolutionary line *Allium* species; **(D)**—the second and **(E)**—the third. Non-conserved AA positions are highlighted as letters and conserved as dots. The whole AA sequence is shown as a normalized alignment logo below. Possible frameshifts are shown as ! and stop codons as *. Damaged sequences are highlighted in peach color in case of frameshift and in purple in case of stop codon. Colored sequences may be assumed to be a pseudogene.

The differences in the nucleotide sequences of the *infA* genes (encoding translation initiation factor IF-1) are undoubtedly correlated with the phylogeny of the *Amaryllidaceae* family. While *Agapanthoideae* and *Amaryllidoideae* species contain genes translated without errors, most *Allioideae*, including *N. bivalve* and most *Allium* species of the first, second and third evolutionary lines, have pseudogenized *infA* (see Figure 4). In some *Allium* species the *infA* genes are completely deleted (for example, in *A. macleanii*). In many species they contain stop codons at different distances from the start of translation (Figure 4). Pseudogenized *infA* genes, containing a stop codon at a short distance from translation start point, are found more often in plastomes of the species belonging to the first and second evolutionary lines. The observed patterns indicate that *infA* genes were pseudogenized multiple times after the separation of the *Allioideae* from other groups within *Amaryllidaceae*.

Analysis of amino acid sequences that lack stop codons and frameshifts in the middle of the sequence has shown that in species of the second evolutionary line there occurs a radical amino acid substitution in Y34S position, while all *Allium* species have such substitutions at I46T. Amino acids of these positions participate in forming rRNA binding sites (nucleotide binding) (Hiratsuka et al., 1989).

#### The *ccsA* Gene Encoding Cytochrome c Biogenesis Protein CcsA

**FIGURE 5:**
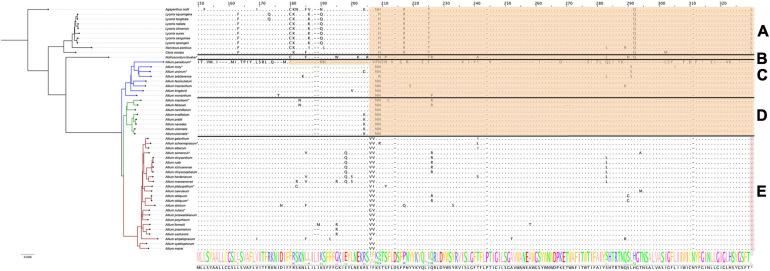
Protein sequence alignment of the cytochrome c biogenesis protein encoded by *ccsA* genes. Àll species are sorted by phylogenetic tree: the first evolutionary line of *Allium* is blue, the second is green, the third is red and the remaining species are black. Species sequenced by our group are marked with *. **(A)** Shows *Agapanthoideae* and *Amaryllidoideae* subfamily species; **(B)** shows *Nothoscordum bivalve* as an outgroup for *Allium* genus; **(C)** shows the first evolutionary line *Allium* species; **(D)**—the second and **(E)**—the third. Alignment here starts from the 150th AA position for better visualization. Non-conserved AA positions are highlighted as letters and conserved as dots. The whole AA sequence is shown as a normalized alignment logo below. Possible frameshifts are shown as ! and stop codons as *. Damaged sequences are highlighted in peach color in case of frameshift and in purple in case of stop codon. Colored sequences may be assumed to be a pseudogene.

The *ccsA* gene is undergoing pseudogenization within all non-*Allium* species as well as in all species of first and second evolution lines (see Figure 5). The plastome of *A. victorialis* (LT699702.1) sequenced in this work was the only exception to this rule, containing normally translated *ccsA*. Interestingly, the other specimen of *A. victorialis* (NC_037240.1) sequenced by Lee et al. (2017) has *ccsA* pseudogenized like other species of the second evolutionary line. We suppose that the plastome of *A. victorialis* (LT699702.1) could contain some inexplicable mistake in the *ccsA* gene and could be ignored when patterns of pseudogenization are discussed. Thus, the *ccsA* gene status (“normal” or “pseudo”) reflects the phylogenetic evolution of the *Amaryllidaceae*: *Agapanthoideae* and *Amaryllidoideae* species as well as basic clades of *Allioideae* form *ccsA* pseudogenes.

Despite the fact that their products are obviously necessary for the normal plant system functioning, the reasons why the *infA*, *rps16*, *rps2*, and *ccsA* genes had lost their functionality remains unclear. It is possible that copies of these genes can be found in nuclear genomes. We checked the sequences of the assembled whole nuclear genome of *A. sativum* which is the only one available in NCBI presently (assembling accession number: GCA_014155895.1) for the presence of the sequences *infA*, *rps16*, *rps2* but gained no positive results. Perhaps these genes can be found in nuclear genomes of other *Allium* species.

### Evolution Rate and Plastome Gene Selective Pressure Analysis

The evolution rate estimation is widely used in phylogenetic and evolutionary studies. Usually pseudogenes and genes with loose selective constraints have the highest rates, followed by positively selected genes. Methods for detecting positive or diversifying selection events are sensitive to evolutionary models, so we treated them as putatively selected only sites, discovered by both MEME and FUBAR methods of analysis (see Supplementary Table 3). The highest rate of evolution, 3,16, was detected in gene *rpl32*. No sites under positive selection are found by MEME and FUBAR in sequence of 59 a.a. length in protein 60S ribosome 32, coded by *rpl32*. We can therefore suppose a neutral model of evolution in most lineages, with selective constraints loosen in some species. Amino acid sequence of *Allium paradoxum rpl32* protein has relatively low homology with sequences of the other *Allium* species and high number of gaps in alignment. In *A. macranthum* and *A. fetisovii* amino acid sequence of 60S ribosome 32 also differs from consensus alignment, so we can either suppose that this protein plays a role in adaptation or rather has lost its selective value in these species.

The next gene in the high evolutionary rate list is *ycf1*, encoding the long protein of 1,761 a.a. with partly known functions and a story of pseudogenization in various plant families. MEME gives 51 sites under positive selection, while only 4 of them coincide with FUBAR list. Selection was detected by AbsRel method in two lineages—*A. mairei* and *A. oschaninii*. We can suggest predominantly neutral evolution of *ycf1* in most lineages, but in some cases we can suppose selective evolution as well. In *A. macranthum*we can see long compensated frameshift, it is an evident trace of stabilizing selection.

The third gene in this list is *psbZ*, encoding a small protein of Photosystem II reaction center. MEME revealed only one site under selection and it does not match with FUBAR data. But the Kn/Ks ratio in *psbZ* is 1,26, one of the highest in all the examined genes. Natural selection obviously affects the sequence, but we cannot detect the particular site of its action. So, we would rather suggest neutral evolution after relaxation of selective constraints (Akashi et al., 2012). The last gene with evo rate >2 is the ndhF gene, coding NADH-plastoquinone_oxidoreductase_subunit_5. We detected it by MEME selection in 15 sites of 734 examined, 4 of them coinciding with FUBAR data. The aBSREL detected positive selection in the ndhF gene in three lineages—*A. macranthum*, *A. forrestii*, and *A. neriniflorum*. Kn/Ks ratio in the *ndhF* gene is 0,28, so we should stay away from the conclusion that selective mode of evolution is significant in all examined lineages, but we may say that in some of them natural selection can play a certain role.

*matK* has an evolutionary rate of 1,98, which is insignificantly lower than 2. MEME has found 13 sites from 521 under selection, and only 3 of them are the same as those found by FUBAR. aBSREL found only one lineage under selection—*A. paradoxum*, and in two species *(A. moly* and *A. macleanii) matK* was pseudogenized. *matK* is widely used as a phylogenetic marker and we agree here that it is reasonable in most cases, but some *matK* sites are still under positive or diversifying selection.

Two genes of small ribosomal proteins (*rps15* and *rps16)* have close evo rates, *rps15* evo rate is 1,68 and rps16 evo rate is 1,52. No selection was detected in rps15 by any method, so we would assume that its evolution is neutral and only negative selection affects it. In *rps16* both MEME and FUBAR detect the same site under selection, so we are not able to argue there is no evidence of selection at all. In some species of the third evolutionary line *rps16* is pseudogenized, but in other lineages it is functional and thus it can be presumably under selection in these lineages.

**FIGURE 6:**
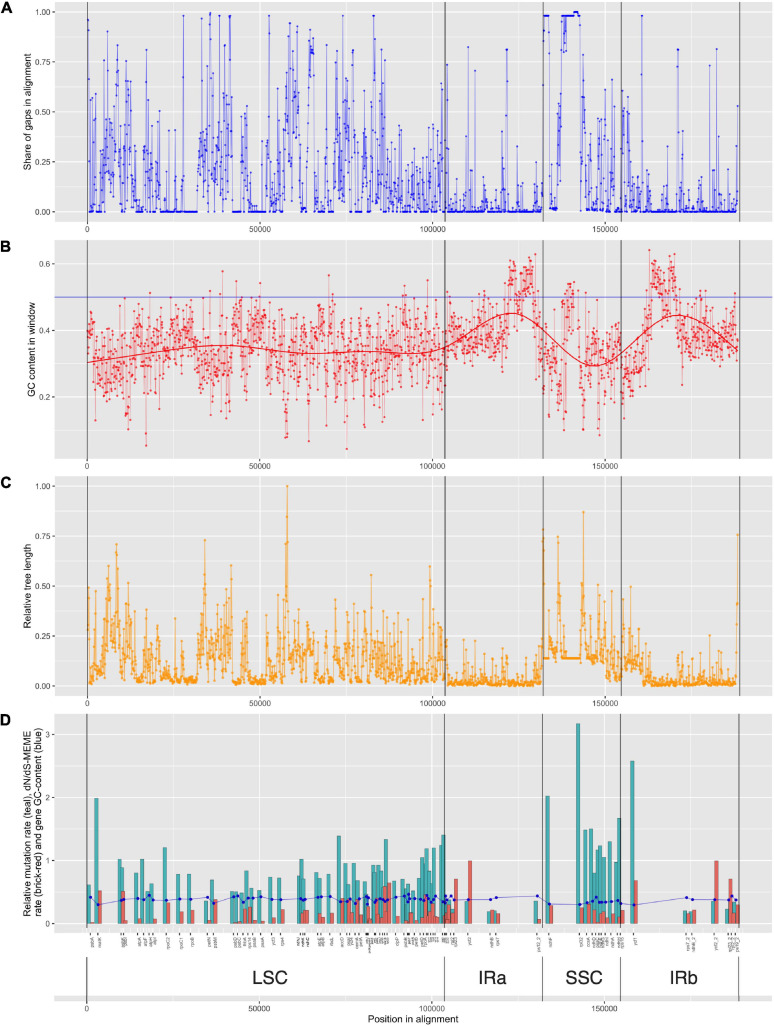
Information on alignment and gene features. Structural components of plastome genomes and genes’ positions are shown [*LSC*, large single-copied region; *SSC*, small single-copied region; *IRa* and *IRb*, inverted repeats **(A,B)**]. **(A)** Share of gaps in alignment in the window of 100 bp. Dots are connected with a line for visibility. **(B)** GC-content in the window of 100 bp. Dots are connected with a line for visibility. A bold line shows regression estimating of GC-content. **(C)** Relative tree length. Dots are connected with a line for visibility. **(D)** Relative mutation rate (teal), Kn/Ks-MEME rate (brick-red) bar-plot and gene GC-content (blue). Dots are connected with a line for visibility.

High percentage of gaps in the small single copy region is very interesting at first glance (Figure 6A), but can be reasonably explained by lineage-specific insertion in *Nothoscordum* and *Narcissus.* Species of genus *Allium* do not have this insertion, so they have many common gaps in alignment.

GC content in different genes varies from 0.29 to 0.45 (Supplementary Table 3 and Figure 6B). Genes with high evolutionary rates usually have lower GC content, e.g., *ycf1* (0,29) or *rpl32* (0,3), whereas genes with low evolutionary rates usually have higher GC content, like *psbN* (0,46) or *atpH* (0,44), but this is not a tight dependence. Genes with low evolutionary rate can also have low GC content, like *psbL* (0,30) (see Supplementary Table 3 and Figures 6B,C). The highest GC content is reported in repeats containing no protein-coding genes (see Figures 6B,D).

We found evidence of positive selection in the *matK* genes (its product takes part in the type II introns splicing), *accD* (the beta subunit of carboxyltransferase, which is part of the plastid acetyl-coA carboxylase), as well as in some *ndh* genes and *petL* (see Table 1). All these genes are involved in electron transport in photosystem II, in particular, in the cyclic transport of electrons. *petL* gene product is cytochrome b6-f complex subunit VI, a component of the cytochrome b6-f complex (Shi et al., 2016). Although only FUBAR results suggest some selection in *petL*, we found it worth mentioning due to its high relevance for cyclic electron transport. The *ndh* genes products are subunits of NADH dehydrogenase and are found to be under positive selection in many angiosperms (for example, Wang et al., 2018). Plants have mechanisms for enduring excess light irradiation using cyclic electron transfer (for example, it is known that mutant tobacco plants with a lost function of the *ndhB* gene demonstrate a greater sensitivity of photosynthesis to the width of stomata opening (Horváth et al., 2000). It is also known that the synthesis of the protein encoded by the *ndhA* gene occurs as a response to photooxidative stress (Martín et al., 1996, etc.). Photosystem II protein M gene *psbM* also has one confirmed site under positive selection. Taking all the above into account, we can assume that adaptive evolution of genes affecting cyclic electron transport around photosystem II, especially encoding the subunits of NADH-plastoquinone oxidoreductase, is supposedly caused by abiotic factors of high altitude regions, mostly by intense light and UV radiation.

**TABLE 1:** Sites under positive of diversifying selection in protein coding genes.

Protein	Gene	Number of sites in alignment	Number of MEME sites under + selection	Number of FUBAR sites under + and − selection	Positions and number of MEME sites, confirmed by FUBAR
Acetyl-CoA carboxylase carboxyltransferase beta subunit	*accD*	480	6	+5 −23	96,156, 176, 477 Total 4
Cytochrome c biogenesis protein CcsA	*ccsA*	330	2	+3 −8	241
Protein TIC 214	*ycf1*	1,761	51	+9 −44	352, 705, 810, 851 Total 4
Hypothetical chloroplast RF21	*ycf2*	2,294	15	+35 −14	474, 475, 595, 691, 1,786 Total 5
NADH-plastoquinone_oxidoreductase_subunit_1	*ndhA*	366	3	+1 −76	206
NADH-plastoquinone_oxidoreductase_subunit_2	*ndhB*	511	1	+2 −12	380
NADH-plastoquinone_oxidoreductase_subunit_4L	*ndhE*	102	1	+1 −13	47
NADH-plastoquinone_oxidoreductase_subunit_4	*ndhD*	507	5	+3 −88	404, 454 Total 2
NADH-plastoquinone oxidoreductase subunit 5	*ndhF*	734	15	+6 −146	299, 510, 514,676 Total 4
NADH-plastoquinone_oxidoreductase_subunit_6	*ndhG*	183	1	+1 −18	34
NADH-plastoquinone oxidoreductase subunit K	*ndhK*	249	3	+2 −20	235, 240 Total 2
Photosystem II protein M	*psbM*	35	1	+1 −1	33
Ribosomal protein L16	*rpl16*	137	1	+1 −22	120
Ribosomal protein L20	*rpl20*	118	2	+3, −3	73
Maturase K	*matK*	521	13	+7 −40	92,324, 345 Total 3
Ribosomal protein S16	*rps16*	83	1	+2 −6	83
Ribulose-1,5-bisphosphate carboxylase oxygenase large subunit	*rbcL*	480	9	+10 −70	91,97, 225, 265 Total 4
RNA polymerase alpha subunit	*rpoA*	341	2	+10 −23	225
RNA polymerase beta subunit	*rpoB*	1,071	9	+5 −158	7, 160, 1,061 Total 3

More support of this assumption came from aBSrel analysis of selected branches. We analyzed the list of aBSrel findings of selection events and the maximal altitudes of habitats and found most species to be referred to as alpine species (occurring at altitudes of 3,000 m and higher), except for two rather lowland species—*A. neriniflorum and A. strictum* (Table 2). We did not find any links between selection events and taxonomic position of the species in the genus. In all branches besides *A. paradoxumndh* genes were discovered to be under selection, only in *A. paradoxum* signatures of selection were found in maturase K gene.

**TABLE 2:** Genes and branches with selection events.

Evolutionary line	Species	Habitat altitude range	Genes shown as being under selection with aBSREL
I	*Allium paradoxum*	No data (lives in shady forests)	*matK*
	*Allium macranthum*	2,700–4,200	*ndhF*, *rpl16*, *rpoC*
	*Allium kingdonii*	4,500–5,000	*ndhK*
II	*Allium neriniflorum*	500–2,000	*ndhF*, *rpoB*
III	*Allium strictum*	No data (lives on open rocks)	*ndhK*
	*Allium przewalskianum*	2,000–4,800	*ndhJ*
	*Allium forrestii*	2,700–4,200	*ndhF*
	*Allium oschaninii*	3,000	*ndhJ*, *rpoB*
	*Allium cyathophorum*	2,700–4,600	*ndhD*, *ndhJ*

Fisher’s exact text for multiple samples provided some more support for hypothesis of selection pressure on *ndh* genes. *P*-value 8.822e-06 allows us to reject H0, some annotations has more selected sites. The only differing annotation is “*ndh* genes,” it differs significantly from “photosynthesis” and marginally from “translation.” Other ontologies do not give significant signal (Supplementary Material 4).

## Discussion

According to our analysis, plastomes in the genus *Allium* evolve predominantly in neutral mode. It is important to remark that only *A. paradoxum* significantly differed from other *Allium* species in having large genomic rearrangements. First of all, major rearrangements occurred in the 4,825 bp inversion (in the region between the *ndhE* and *rpl32* genes in SSC). *A. paradoxum* also demonstrates complete loss or pseudogenization of all its *ndh* (NADH dehydrogenase-like) genes. It was shown that *A. paradoxum* has the shortest known plastome of *Allium* species due to a large number of small deletions (145,819 bp). All the NADH-dehydrogenase complex genes (*ndh* genes) were found defunctionalized in the *A. paradoxum* plastome. This event may be supposedly associated with its adaptation to specific environmental conditions, rather uncommon among the species of *Allium* (shady humid forests). Some of *A. paradoxum* plastome genes have also lost their functionality in contrast to the rest of the studied species: *rpl22* was deleted and *rpl23* underwent pseudogenization (Omelchenko et al., 2020). We found that all other *Allium* species of the first evolutionary line had neither major rearrangements nor significant differences in the number of genes compared to *Allium* species of two other lines. The results comparing *A. paradoxum* and *A. ursinum* were published (Omelchenko et al., 2020).

Plants have evolved a suite of adaptive responses to cope with variable environmental conditions. Ranges of *Allium* species are wide not only regarding latitude and longitude, but also altitude. Numerous species occupy mountain territories, budding high altitude species from each evolutionary line (Friesen et al., 2006). That is why putative adaptations to specific high altitude environments were of our special interest. Natural areas of species habitats are different in climatic parameters in local areas. Considering temperature or humidity we can only speak of parameters that are more or less typical for the habitat of a particular species. But maximal altitudes as well as the total amount and spectrum of sunlight are detected more precisely and we can consider it as a limiting factor that could direct natural selection.

Some genes with discovered sites under positive selection—*rpoA*,*rpoB* and *rbcL*—code essential enzymes for chloroplast protein synthesis (*rpoA* and *rpoB)* and sugar synthesis (*rbcL)*. *RbcL* gene evolves rapidly in many clades (see, for example, Sen et al., 2011; Galmés et al., 2014; Gao et al., 2019), and there is some data that its adaptive evolution may promote successful colonization of high altitude habitats (Zhao et al., 2019). The *rbcL* and *rpoC2* genes were involved in adaptation of *Cardamine* species to high or low altitudes. Authors of the study speculated that amino acid residues found to be under positive selection in RUBISCO could possibly be involved in the modulation of RUBISCO aggregation/activation and enzymatic specificity (Hu et al., 2015). Also the *rbcL* is under selection pressure in shade-tolerant *Oryza* species (Gao et al., 2019).

Other genes of those that were found under positive diversifying selection in *Allium* genus in this work were also found under selection pressure in shade-tolerant *Oryza* species, namely *accD* (Gao et al., 2019) and in *Cardamine* species adapted to different altitudes, namely *accD*, *ccsA*, *ycf1,rpl20* and *matK* (Hu et al., 2015). The *matK* gene also deserves a separate discussion. This gene is found under positive selection in our analysis of maturase K protein sequence in 3 sites from 521. The *matK* gene is under positive selection in many clades and its role in splicing of type II introns may also play a role in adaptation. Nevertheless, Kn/Ks ratio of *matK* is rather low (0,76) and we cannot be sure it is under selective pressure in the course of *Allium* adaptation to a wide range of environments, characteristic for this widespread genus.

Our analysis revealed that many genes from the *ndh* family are under positive selection—*ndhA*, *ndhB*, *ndhD*, *ndhE*, *ndhF*, *ndhG*, and *ndhK*. *PetL* is found only by FUBAR, but we are considering it here as well because its product is involved in the same biological process. All these genes play certain roles in electron transport across the PSII and especially in cyclic electron transport. PETL codes a component of the cytochrome b6-f complex (Shi et al., 2016) and genes from *ndh* family code subunits of NADH-dehydrogenase. We know that loss-of-function mutants of *ndhB* gene in tobacco demonstrate sensitivity of photosynthesis to moderate stomatal closure (Horváth et al., 2000) and *ndhA* protein is synthesized in response to photooxidative treatment (Martín et al., 1996). *NdhK* is a part of NADH-oxidizing subcomplex (Elortza et al., 1999) and is positively selected in some angiosperm clades (Wang et al., 2018), in particular in species adapted to contrasting high/low altitude habitats (Hu et al., 2015) and shade-tolerant and sun-loving species (Gao et al., 2019).

Cyclic electron transport across PSII is activated in response to intense light or when stomata are closed to prevent production of reactive oxygen compounds and photodamage of PSII and PSI. Under excess light and/or closed stomata (CO_2_ deficiency) plastoquinone pool regenerates and thereby a regeneration of NADP + occurs. NADPH cannot be expended in Calvin cycle in case of CO_2_ deficiency. Substrate for ferredoxin-NADP-reductase is deficient and excess of excited electrons can cause membrane oxidation, generation of active oxygen species and damage of photosystems. To prevent oxidative stress, plants start cyclic electron transport.

Genes from the *ndh* family are strongly overrepresented in the selected gene list in our study—we found sites under selection in 7 genes of 11 of this family. The most plausible explanation of this fact is their relevance in adaptation to conditions of high light intensity. Taking into account pseudogenization of *ndh* genes in *A. paradoxum*, we can accept this hypothesis as the first choice. Data from Fisher’s exact test provide moderate support to this hypothesis. *Ndh* is the only ontology that gives significant difference in ratio of selected to unselected sites and not in all comparisons. If we can speak of signatures of selection in this study, we can mention only genes from *ndh* family.

High altitudes have specific climate parameters, namely temperature extremes, strong winds, high solar radiation and lower atmospheric pressure (Rangwala and Miller, 2012). All these conditions affect leaf temperature, gas exchange constants, vapor pressure deficit, Michaelis-Menten constants for carboxylation and oxygenation and some other parameters affecting photosynthesis (Wang et al., 2017). Each extra 1,000 m in altitude makes photosynthesis more effective because of enhanced photosynthetic photon flux density and at the same time more problematic because of decreasing χ, which is a decreasing function of the leaf-to-air vapor pressure deficit (VPD) (Wang et al., 2017).

In mountain habitats with their fast circadian changes of light intensity and humidity and, consequently, with long periods of light phase of photosynthesis going on with closed stomata, cyclic electron transport is essential for preventing photodamage of PSII and PSI. In some plants high contents of phenolic chemicals play a role in preventing photodamage without switching to cyclic electron transport, and in *A. ursinum* high concentration of polyphenolic compounds was indeed reported (Lachowicz et al., 2017; Tóth et al., 2018).

Oxidative stress may affect not only electron transport, but RNA synthesis as well. In *Mycobacterium tuberculosum* mutations in *rpoB* gene can modulate bacterial survival in high oxygenic environments inside macrophages (O’Sullivan et al., 2005). In *Escherichia coli* cells, growing in aerobic conditions, the guanine base is oxidized to 8-oxo-7,8-dihydroguanine, which can pair with adenine as well as cytosine. So fidelity of RNA synthesis depends upon proper work of RNA polymerases and substitutions in its β and β’ subunit, coded by *rpoB* and *rpoC* genes are under selection in aerobic conditions (Inokuchi et al., 2013). Based on these facts we can hypothesize that highly oxygenic conditions in mountain habitats can cause selective pressure upon RNA polymerase function in chloroplasts, changing its protein sequence to establish stable function in conditions characterized by an excess of active oxygen, produced by overloaded Photosystem II.

So we can conclude that species of genus *Allium* appear to use various possible ways to prevent photodamage, but at high altitudes maintaining cyclic electron transport is most essential.

## Data Availability Statement

The datasets presented in this study can be found in online repositories. The names of the repository/repositories and accession number(s) can be found in the article/Supplementary Material. The custom scripts used in this study for figures preparation have been deposited in Github (https://github.com/nikitin-p/Allium_analysis).

## Author Contributions

VS, AK, and AS: conceptualization, ideas formulation, or evolution of overarching research goals and aims. IA, PN, MB, EK, and AES: software and bioinformatics analysis. VS, IA, AK, DO, ML, and AS: investigation, conducting a research and investigation process, specifically performing the experiments, or data/evidence collection. MA and SK: resources and provision of plants. VS, PN, and AS: data curation, management activities to annotate (produce metadata), scrub data, and maintain research data (including software code, where it is necessary for interpreting the data itself) for initial use and later reuse. VS, IA, PN, AK, and AS: writing—original draft preparation, creation and/or presentation of the published work, specifically writing the initial draft (including substantive translation). ML: writing—review and editing. PN: visualization and preparation of the figures. AS: project administration, management and coordination responsibility for the research activity planning and execution, funding acquisition, and acquisition of the financial support for the project. All authors contributed to the article and approved the submitted version.

## Conflict of Interest

The authors declare that the research was conducted in the absence of any commercial or financial relationships that could be construed as a potential conflict of interest.

## Footnotes

1 https://plant.depo.msu.ru/
